# Spark or Sound: How Two Differing Explanatory Strategies Impact the Debate on the Physical Nature of Neuronal Excitability

**DOI:** 10.3390/membranes16050172

**Published:** 2026-05-08

**Authors:** Benjamin Drukarch, Micha M. M. Wilhelmus

**Affiliations:** Department of Anatomy and Neurosciences, Amsterdam Neuroscience, Amsterdam UMC, Vrije Universiteit Amsterdam, De Boelelaan 1117, 1081 HZ Amsterdam, The Netherlands; b.drukarch@amsterdamumc.nl

**Keywords:** neuronal excitability, action potential, bioelectricity, thermodynamics, non-linear acoustics, perspectival realism, scientific explanation

## Abstract

Neuronal excitability manifests itself mainly in the form of non-linear, self-regenerative waves of electricity moving along the surface of neuronal axons. These waves are commonly known as action potentials (APs). Theoretical and experimental investigations of the physical and functional characteristics of APs have broadly followed along the lines of the ionic hypothesis and the associated mathematical model introduced by Hodgkin and Huxley (HH). In the current form of this bioelectrical framework, adopted in mainstream physiology and other biological sciences, the axonal membrane is conceptualized as an electronic circuit where electric current is generated and propelled as a result of the time-dependent opening and closure of voltage-operated ion channel proteins, allowing passive flow of specific ions across and along the membrane, powered by their respective electrochemical gradients. Although representing mainstream research, the bioelectric perspective has been criticized for its narrow focus on the electrical characteristics of APs, whilst ignoring other physical manifestations of the nerve signal, particularly mechanical and thermal changes coinciding with AP propagation. As an alternative, a macroscopic thermodynamics-based acoustic theory has been outlined, in which all electric and non-electric manifestations of the nerve signal are considered as a result of a single density pulse in the axonal membrane carried by a reversible lipid membrane phase transition and momentum conservation. Representing a minority view, however, this unified, acoustic perspective on the physical nature of neuronal excitability is largely ignored by representatives of the bioelectric perspective. Here, we draw special attention to the philosophical dimension of the communication failure between the two communities of scientists. We argue that adherents of the bioelectric perspective favor a mechanist type of explanation, whilst supporters of the acoustic perspective are committed to so-called covering-law types of explanation. We conclude that it is this thus far unrecognized philosophical rift, rather than specific scientific differences in opinion, that blocks fruitful interdisciplinary cooperation necessary for building a comprehensive, fully integrated notion of the physical nature of neuronal excitability. Suggestions of how to bridge this conceptual gap are formulated.

## 1. Introduction: Formulating the Nature of the Problem

*“At the heart of science is an essential balance between two seemingly contradictory attitudes- an openness to new ideas, no matter how bizarre or counterintuitive they may be, and the most ruthless skeptical scrutiny of all ideas, old and new”.*

*C. Sagan in The Demon-Haunted World: Science as a Candle in the Dark.*

Excitability is a universal physical phenomenon observed in a large variety of (biological and non-biological) dynamical systems. Although these systems differ considerably in molecular detail, they do share characteristic features of excitable media. These include, most particularly, the capacity to support propagation of uniform waves of some sort and a stable resting state, where a small perturbation is rapidly damped out but a suprathreshold disturbance makes a large, i.e., non-linear, excursion before the system returns to its resting point, typically accompanied by a refractory period, in which the system temporarily becomes immune to repeated stimulation, and annihilation of pulse waves upon collision [1,2]. This generally accepted description of the macroscopic physical characteristics of excitability in dynamical systems differs considerably from its colloquial use in biology and psychology, where it is commonly taken to describe the ability of cells or other complex biological systems not only to sense but also respond in a case-defined manner to any external, physical, chemical, or other disturbance they are exposed to. Considered in this somewhat broader sense, cellular excitability is, for instance, sometimes taken as the natural, i.e., physical, basis of fundamental life processes, including sentience and consciousness [3,4]. Thus, standing at the crossroads between physics, biology, and psychology, where it represents a core concept in teaching and research, the study of the physical basis of excitability in biological systems has the potential to inform important biological and/or psychological phenomena.

Neurons are perhaps the most extensively studied example of an excitable biological system. In neurons, excitability, defined in its classical, i.e., physical, sense, is traditionally linked to the generation and propagation of non-linear (so-called all or none) self-regenerative waves of electrical activity along the surface of the axonal membrane following electrical stimulation. Such waves, commonly known as action potentials (APs) or spikes, are thought to ensure the fast and reliable transport of binary-coded information between neurons and other excitable cells, and as such, the electrical AP concept forms one of the pillars of the modern understanding of neuronal physiology and function [5]. However, does the definition of APs as a non-linear electrical wave phenomenon warrant the conclusion that neuronal excitability itself is (only) (bio)electric in nature and should not only be defined but also explained, understood, and modeled accordingly? Indeed, historically, the fact that electricity was the primary source of energy used to elicit APs in nerves and muscles was taken by most to indicate that the two phenomena were identical or, at least, shared defining characteristics [6,7]. Not everyone agreed though (e.g., [8]), and the matter remained undecided until the middle of the 20th century, when the so-called Hodgkin and Huxley (HH) theory and mathematical model of electrical conductance across the axonal membrane appeared to have finally settled the issue about the physical nature of neuronal excitability [9]. However, in a set of recent papers, we presented and commented on a large body of published experimental results and theoretical considerations that (continue to) cast doubt about the completeness and/or scientific robustness of the electrical circuit-based framework for neuronal excitability formulated by HH [10,11,12]. As discussed by us, largely ignoring this repeatedly voiced critique but supported and extended by the joint efforts a broad forum of (other) eminent electrophysiologists, molecular and cellular (neuro)biologists, (neuro)biochemists, and computational (neuro)scientists, over time, the HH theory and model evolved to become the current “received”, i.e., textbook, explanation of the molecular and cellular basis of excitability in neurons [13,14,15,16]. In contrast, representing the view of a small minority group of theoretical (bio)physicists and physical–chemistry-oriented physiologists and building primarily on the abstract and (often) difficult to understand fundamentals of (macroscopic) thermodynamics [11], the ideas and arguments put forward by these dissenting scientists, although meant to engage representatives of mainstream neurophysiology (and other neuroscientists) into discussion and (re)investigation of the (overall) scientific validity of their (bio-electricity-only based) conception of neuronal excitability, misfired and were, in fact, misconceived and misconstrued as an unwarranted threat to the ruling theory and model [17,18]. As a consequence, to the likely dismay of those who recommend an open-minded, interdisciplinary approach as instrumental for progress in our understanding of neuronal physiology and function (e.g., [17,19,20,21]), the sought-for (interdisciplinary) interaction and cooperation to stimulate joint development of a (more) comprehensive, integrated physics-based conception of neuronal excitability failed to take off.

Here, we take our historical and conceptual analysis of this important case in modern (cellular) neurobiology and physiology a step further by arguing that this example of (thus far) failed interdisciplinary interaction and communication between groups of scientists, rather than being the result of an irreconcilable difference in opinion about the validity and interpretation of experimental results or the specific claims of scientific theories, is rooted primarily in divergent views of what a valid scientific explanation in general should necessarily entail and by what criteria it should be evaluated between two communities of scientists operating within the boundaries of two different scientific perspectives on neuronal signaling. More specifically, we claim that scientists adhering to what we have previously called the bioelectric perspective [22], representing the current majority view, over time have become committed to what is known as mechanistic types of explanation, whereas scientists supporting a non-linear acoustic-based approach, i.e., the minority view, strive to formulate a non-mechanistic covering-law version of explanation. Drawing from similarities with an extensively documented case in stem cell research [23,24], we conclude that recognition of and dedicated efforts to bridge this gap between theoretical, i.e., philosophical, commitments and explanatory strategies “traditionally” directing research efforts in different communities of scientists will be instrumental to secure productive forms of cooperation across perspectives necessary to solve outstanding questions about the physical nature of neuronal excitability and its relationship to phenomena of biological and/or psychological interest.

## 2. Explaining the Physics of Neuronal Excitability: The Bioelectric Perspective

Explanations are counted amongst the most distinctive products of any scientific (sub)discipline, and the explanation of natural phenomena in a testable form is considered one of the main tasks of science. Relying on the earlier description of a scientific field by Darden and Maull [25], for the purpose of the current paper, a scientific discipline or field is thought to center around a central “problem” and consists of a domain of items accepted as facts related to that problem, with general explanatory factors and goals providing expectations as to how the problem is to be solved, techniques and methods, and sometimes, concepts, laws, and theories, which are related to the problem, all of which are directed to realize the field’s explanatory goals. Accordingly, scientific explanations should be considered as products of scientific inquiry, constructed with a set of tools and in accordance with the norms of a scientific discipline on which the status of “explanation” has been bestowed by (the) members of the respective discipline [26].

From its gradual inception as a separate scientific (sub)discipline during the early decades of the 19th century, following the early lead of Galvani and his immediate successors, neurophysiology, as a branch of physical biology, focused on describing and characterizing the physical nature of neuronal excitability as an electric phenomenon [27]. To reach this goal, stimulated by the developing general scientific, industrial, and public interest in electric phenomena and technology, neurophysiology used and adapted theoretical concepts, metaphors, and models from electronics and electrochemistry, conceived experiments, and developed experimental equipment suited exclusively to probe the electrical manifestations of the nerve signal [10]. Building on the apparent success of this line of research to study and catch the nerve signal in electric terms, (Anglo-Saxon) neurophysiologists introduced the term AP to describe its perceived primary characteristics and direct (further) investigation into underlying mechanism(s) and effects [10]. Attesting to its lasting impact, current neurophysiological and even neuroscientific knowledge and understanding in general are claimed by some to be fully built on the concept that “Electrical signaling underlies the development, plasticity, and pathology of all sensory, motor, and cognitive functions, and analyses of the nervous system rely on electrical activity (directly for electrophysiology and EEG, indirectly for calcium imaging and fMRI).” [28]. As such, electrical signaling, primarily manifesting in neurons in the form of APs, constitutes the current cornerstone of scientific (and public) explanation, understanding, and modeling of neuronal function from the molecular to the systems level [29]. As a consequence, both for specialists in the field and informed lay people, neurons and, in fact, the whole nervous system, including the brain, are commonly thought to “run on electricity”. It is, therefore, not surprising that neuronal excitability, defined as the ability of neurons to generate and propagate non-linear waves of electricity known as APs, is considered to be the primary (biophysical) characteristic of nerve cells, detailed and continued investigation and physical characterization of which will prove essential for further progress in the scientific explanation and understanding of neuronal physiology and function.

**Figure 1:**
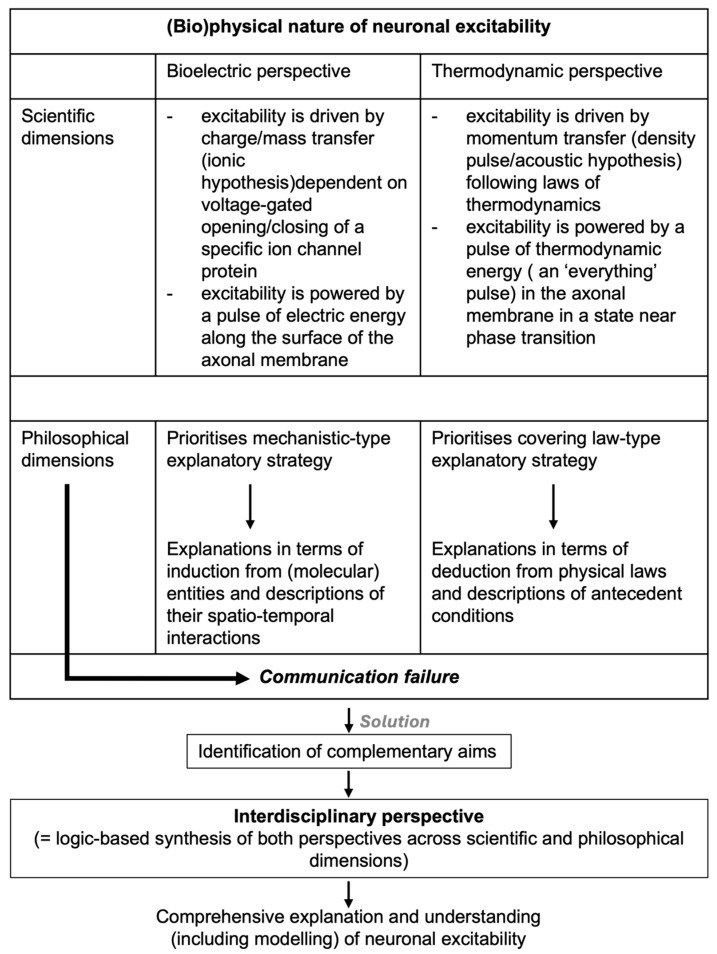
Schematic overview of the scientific and philosophical dimensions of the debate about the physical nature of neuronal excitability.

In this highly successful line of biophysical research, over a period of approximately 100 years, three generations of (cellular) neurophysiologists played a leading role [30]. Their joint efforts ultimately culminated in the introduction of the HH model, celebrated by some as “the most important model in all of the physiological literature” [31]. Acclaimed as perhaps the biggest achievement of the discipline, the HH model has proven vital for the further development of (neuro)physiological theorizing and experimenting along the line(s) of the so-called “animal electricity paradigm” [15]. In a recent reconstruction of the final path leading to the current, i.e., bioelectric, explanation of AP physics, neurophysiologists [32] summarize how HH, in their quest to build a mathematical, physical theory-informed model of the electrical characteristics of the AP, first designed an impressive set of experiments using recent innovations in biophysical technology (i.e., the voltage-clamp technique) in combination with a newly introduced neuronal preparation (i.e., the squid giant axon). Following up on some earlier work, whilst developing their fully electrical framework, HH applied the voltage-clamp technique on the squid giant axon preparation to test if voltage-sensitive Na^+^ and K^+^ permeability changes are both necessary and sufficient for the generation of APs. In doing so, HH, indeed, established that electrical depolarization of the axonal nerve membrane produces two effects: an early influx of Na^+^ into the axon, followed by a delayed efflux of K^+^ from the axon’s interior. In fact, by varying the external Na^+^ concentration and tracing the flux of radiolabeled K^+^ across the axonal membrane in relation to AP kinetics, HH achieved a major experimental breakthrough in decomposing membrane currents under voltage-clamp into fully separate components carried by Na^+^ and K^+^ ions, both of which are controlled by membrane voltage. This novel concept became known as the “ionic hypothesis” (Figure 1), which postulated that excitability relies on the controlled movement of ions across the cell membrane, altering the electrical potential difference between the inside and outside of the cell [33,34]. Moreover, using cable theory and assuming that the AP travels along the axonal surface at a constant speed independent of voltage [35], HH developed their mathematical model further into a differential equation that quantitatively describes the ionic currents during AP propagation. Modeling the propagating AP with this equation, HH successfully reproduced key characteristics of the electric AP moving in the squid axon, including its form, amplitude, velocity, and refractory period [9]. For this, as recounted by Hille [32], HH spent two years analyzing all the results of their experiments, building a kinetic model using these results, calculating axon responses, and publishing the set of five papers that would revolutionize the neuroscientific understanding of the AP and firmly establish the currently accepted view of the electric nature of neural excitability. However, although their 1952 papers successfully introduced a formal kinetic model of the propagating AP, at the time, HH could only speculate about the molecular mechanism(s) responsible for the voltage- and time-dependent changes in current flow across the axonal membrane accompanying this electric phenomenon. In doing so, HH proposed that the ionic conductances were controlled by hypothetical charged “particles” within the membrane that were driven toward one side or the other by the changing local electric field [32]. Despite being cleverly used to derive important parameters, allowing HH to fit their model to the result(s) of their studies, the physical nature of these particles and that of NA^+^ and K^+^ permeation across the axonal membrane, whether by separate pores, carriers, or other more diffuse entities, however, remained to be elucidated. In fact, in their original paper, HH pointed out that the apparent success of their model (agreement between equations describing conductance changes and their voltage clamp data) “must not be taken as evidence that our equations are anything more than an empirical description of the time-course of the changes in permeability to sodium and potassium.” In fact, HH explicitly emphasized that “An equally satisfactory description of the voltage clamp data could no doubt have been achieved with equations of very different form, which would probably have been equally successful in predicting the electrical behavior of the membrane”. Thus, according to HH, although “certain features of our equations were capable of a physical interpretation, but the success of the equations is no evidence in favor of the mechanism of permeability change that we tentatively had in mind when formulating them” [9]. Indeed, identification and (molecular) characterization of the proposed pores as separate, but functionally similar, ion channel proteins, regulating the flow of Na^+^ and K^+^ ions across the axonal membrane, had to await theoretical and, in particular, major technological developments from the 1970s onwards in a number of related scientific disciplines, including molecular biology, biochemistry, and structural chemistry, in combination with introductions into the neurophysiology of a refinement of the voltage-clamp technique, i.e., the patch-clamp, which allowed the recording of ionic currents on very small patches of membrane (for historic reconstruction, see [10] and the references therein). Despite continued and vehement opposition to (parts) of the ion channel protein concept and its proposed causal role in the AP phenomenon from “old school” physical–chemistry-oriented neurophysiologists, like Ichiji Tasaki and Gilbert Ling [36,37] and, more recently, Hirohisa Tamagawa [38], over time, the combined, i.e., interdisciplinary, efforts by scientists from these fields led to the full acceptance and understanding by neurophysiologists—and, indeed, across mainstream neuroscience as a whole—of voltage-gated ion channels as molecular entities [39]. As such, the (voltage-gated) ion channel protein concept provided a sophisticated, i.e., molecular mechanistic, foundation for the electrical events observed during AP generation and propagation in neurons (and other excitable cells), which had not been possible at the time of HH’s original, i.e., primarily biophysics-based, theoretical and experimental work.

Positively acknowledging the framework introduced by HH and supported by the large majority of their colleagues, “modern day” textbook explanations of the AP phenomenon in neurons usually develop the idea that, upon electrical depolarization of the axonal membrane above a certain threshold, dedicated voltage-gated Na^+^-ion channel proteins present in the membrane open, allowing Na^+^ ions to passively diffuse into the axon, driven by their electrochemical gradient over the membrane. After that, the Na^+^-ion channel proteins transition to an inactive state, ending the inward diffusion of Na^+^-ions. Parallel to this inactivation of Na^+^-ion channel proteins, separate voltage-gated K^+^-ion channel proteins, also located in the membrane, open, allowing passive diffusion of K^+^-ions out of the neuron by their electrochemical gradient. Because the K^+^-ion channel proteins return only slowly to their closed state, the membrane first hyperpolarizes, followed by a return to the resting state potential. In this exclusively bioelectric perspective, although discharge of the membrane capacitor via channel-mediated transport has also been proposed to contribute to passive conditions, propagation of the AP is classically explained by assuming that some of the local current in the axon, generated by the inward flow of Na^+^-ions during AP initiation, spreads passively along the inside of the axon (until the current leaks out of the axon through the membrane), sufficiently depolarizing the membrane at a neighboring segment of the axon to cause local opening and subsequent inactivation of Na^+^-channel proteins, followed by opening and closure of K^+^-ion channel proteins, in a process that then repeats itself while the AP moves forward along the surface of the axon as a self-regenerative wave of bioelectricity, similarly to a “burning fuse of gunpowder” [40] (Figure 1). However, it is important to note that this bioelectric perspective, in which the membrane is idealized as a “sieve” acting like a filter that allows some ions to cross and not others and for which its molecular features (i.e., the presence of voltage-gated ion channel proteins) enable it to change its selective permeability upon excitation [41], is not the only possible description of a propagating non-linear axonal (electric) pulse and only addresses its membrane voltage-related aspects (see next section). Moreover, although expression of voltage-gated ion channels may be a necessary requirement for APs to be generated in excitable cells under physiological conditions, their presence is not sufficient to make any living cell excitable.

Less noticed perhaps, but particularly important for the ensuing discussion—because of the combination of its apparent descriptive success, remarkable predictive power and smooth alignment with the mechanistic standards and expectations of explanation developed in modern experimental biology and related life sciences [42,43]—is the interdisciplinary effort of neurophysiologists, molecular biologists, and biochemists to identify and characterize the electrical nature of the AP in molecular terms. They in general succeeded in turning large segments of neurophysiology from a primarily (bio)physics-oriented discipline—that is, a “top-down” non-reductionistic discipline focusing on formulating explanations via quantitative, physico-chemical theory-guided analysis of the biological problem under study [44]—into a reductionistic, i.e., “bottom-up”, biomolecular science, in which higher level complex biological phenomena like the AP are explained in terms of lower-level causal, mechanistic relationships between real molecular entities, i.e., its component parts, and their spatiotemporal interactions (Figure 1), rather than by referring to physical and/or chemical laws [45]. Importantly, in this mechanistic approach to scientific explanation, it is a complete and accurate description of the interacting but separate parts working together that does the explaining. In other words, mechanistic explanations are constrained by the norm of the accurate representation of real components and causal relations between these components and their organization, in which there is no (specific) need for laws or general principles [45,46]. As argued by Carl Craver and other supporters of the neo-mechanist school of thought in current philosophy of science, it was, in fact, this “molecular turn” in (cellular) neurophysiology, with its emphasis on identification and functional characterization of separate ion channel molecules, that eventually allowed for the broadly welcomed “upgrade” of the original HH model from a “merely” phenomenological, physical, and chemical law(s)-derived description and “how-possibly” explanation sketch to a “how-actually”, i.e., full mechanistic, explanation, as favored in contemporary experimental biology and neuroscience [42,47]. Thus, the lasting popularity of the modern electric framework introduced by HH in neuroscience in general, and neurophysiology in particular, should be understood against the background of its apparent, almost unique, ability to “catch” a complex “macroscopic” biological phenomenon (i.e., the propagating AP) in a mathematical formalism describing mesoscopic events (i.e., the voltage- and time-dependent flow of ions carrying current across and along the axonal membrane) enacted by the respective opening and closure of separate classes of microscopic (molecular) entities (i.e., the voltage-dependent Na^+^- and K^+^-ion channel proteins, respectively). At the same time, however, the warning issued by Alan Hodgkin himself in the early 1960s—that “In thinking about the physical basis of the action potential perhaps the most important thing to do at the present moment is to consider whether there are any unexplained observations which have been neglected in an attempt to make the experiments fit into a tidy pattern …” [48]—was not followed up by mainstream, i.e., mechanism-oriented, neurophysiologists. More than skepticism about the ion channel protein concept as such, it was this concern of Hodgkin which motivated “old school” neurophysiologists like Tasaki and others to keep scrutinizing the very mechanistic underpinnings of the AP phenomenon put forward in the bioelectric perspective, identifying gaps in the prevailing interpretation of data and searching for alternative explanations.

## 3. Explaining the Physics of Neuronal Excitability: The Acoustic Perspective

In their book *Molecular Basis and Thermodynamics of Bioelectrogenesis*, Schoffeniels and Margineanu [49] argue that it may be typical human behavior to (almost) forget experimental results that defy integration into an otherwise very successful explanation of a natural phenomenon. This seems a very apt description of what (almost) happened to the (largely) reversible temperature changes during AP propagation that had been reported on from the early 20th century onwards but remain(ed) difficult to (adequately) reconcile with the electric conductance-based framework for excitability proposed in the HH model. In fact, it was these earlier experimental data that Hodgkin referred to as an example of “unexplained observations which have been neglected” [48]. Indeed, building on the voltage- and time-dependent opening (and closure) of selective ion channel proteins operating as resistors in a (virtually passive) lipid membrane, which in their model is considered as a mere insulator that acts as a capacitor with constant capacitance, HH’s ionic theory, being dissipative in nature, could only fairly accurately account for the initial release of heat from the nerve cell observed during AP propagation, but not for its subsequent (partial) reabsorption as the signal passes by. If proven correct, therefore, the reversible temperature changes accompanying AP propagation would suggest that neuronal excitability, rather than representing a self-regenerative but irreversible wave of electrical energy relying on the transfer of mass (in the form of charged ions) leading the wave front, as per the HH theory, instead rests on a (spontaneously) reversible physical phenomenon alike, for instance, a mechanical wave of some sort. Before Hodgkin, the importance of addressing the issue of net heat production (or absence thereof) during AP propagation for answering fundamental questions concerning the physical nature of neuronal excitability had been considered by Hill [50,51,52], who concluded the following pointedly: “Why did people go on trying to measure the heat production of nerve, in spite of repeated failure? Chiefly, I suppose, in order to settle the question of whether the nerve impulse is the sort of physical wave in which the whole of the energy for transmission is impressed on the system at the start.” [52].

Reversible temperature change, however, was not the only “almost forgotten” experimental challenge facing the bioelectric perspective. Thus, already decades before the first publication of the HH model in 1952, it had been noted that nerve activation not only induces an electrical pulse but also elicits a mechanical contraction of the nerve itself [53]. From the 1960s onwards, facilitated by improvements in experimental techniques and model preparations, it was such unexplained and largely ignored observations about non-electrical physical manifestations of neuronal excitation coinciding with AP propagation that led “old school” neurophysiologist Ichiji Tasaki and coworkers to initiate a physical–chemistry-based research program that aimed to provide a clear, i.e., physically acceptable, account of the role(s) played by all the observable physical processes manifested alongside the electrical AP [37]. In doing so, the work of Tasaki’s group not only succeeded in confirming the reversible nature of neuronal heat release during AP propagation [54,55] but also demonstrated that AP propagation is accompanied by mechanical changes in the form of swelling and subsequent shrinking and shortening of nerve cells [56,57,58]. Moreover, the work of Tasaki and other investigators also served to draw attention to the absence in the HH model (and its successors) of well-defined environmental parameters. These include temperature, pH, or extracellular viscosity, all of which are known to affect key characteristics of the AP, like the resting potential and pulse amplitude, duration, and velocity of propagation [59,60,61]. In addition, the outcome of other, often technically sophisticated, experiments casts doubt about the exclusive role of Na^+^ and K^+^ ions in maintaining neuronal excitability [62,63,64,65]. Nevertheless, the mechanical (and other non-electrical) manifestations of the AP detected by Tasaki et al. (and others later on, e.g., [66,67,68,69,70]) were not necessarily in (fundamental) disagreement with the mechanistic explanation offered by the HH model (they are “just” not taken into account in it; see for instance, Rvachev [71] and El Hady and Machta [72], who independently proposed to link the thermal and mechanical changes to the electric framework through additional equations; see, however, Rvachev and Drukarch [73] for a critical comparison of these two “extended electrical AP” models). Even more so, because Tasaki’s research was not concerned with the identification and functional characterization of the workings of separate molecular components and their interactions but instead looked for explanations of AP manifestations at the level of the collective, i.e., macroscopic and thermodynamic, behavior of the (macro)molecular constituents of the axonal membrane and submembranous cytoskeleton operating as one interconnected biological interface (for a recent overview, see Drukarch and Wilhelmus [12] and the references therein), his work was largely overlooked (and forgotten) by the majority of modern day, mechanism-oriented, experimental neurophysiologists.

However, at the start of the current millennium, contemplating a radical departure from the generally accepted mechanistic molecular framework [74], Tasaki’s physical and chemical theory-guided work did provide the inspiration for two groups of (membrane) biophysicists to (again) challenge the (current form of the) HH theory and model, arguing that it is “incapable of explaining or predicting many experimentally observed characteristics of nerve signal propagation” [75]. More specifically, instead of providing a mechanistic explanation of the (electrical) AP phenomenon in terms of the opening and closure of specialized, microscopic protein components expressed in an otherwise passive impermeable lipid membrane barrier, these scientists sought to develop a thermodynamics-based, first principles-derived macroscopic account, in which neuronal excitability is controlled by the collective thermodynamics-based properties of the membrane interface consisting of lipids, proteins, ions, water, etc. For the development of their thermodynamic perspective on cellular excitability, they drew heavily from the work of theoretical physicist Konrad Kaufmann in the late 1980s. In his work, Kaufmann applied Albert Einstein’s thermodynamic treatment of interfaces to biological systems and used it to formulate a new theory of neuronal excitability that strived to capture the various (established and predicted) physical manifestations of the AP wave phenomenon in a single, i.e., unified, and consistent framework derived from physical principles (for extensive reviews, see [11,76]).

Somewhat different from Tasaki, however, who, especially in his later (theoretical and experimental) work on neuronal excitability, focused on ion-induced volume-phase transitions in polyelectrolyte gels [12], for the development of their thermodynamic theory-guided account of the AP, Kaufmann and his followers identified the axonal membrane interface as the system of interest. Importantly, because this interface is to some degree “decoupled” from its surroundings, it has its own thermodynamic states and properties [76]. Although disputed by some as to the proper interpretation of its results, experimental support for this choice of system came from an earlier study by Terakawa and Nakayama [77], who demonstrated that APs can still be excited in axons after the removal of intracellular material. This was taken to suggest that APs, in contrast to the HH theory, propagate in the axonal membrane itself. Moreover, the experimental observations concerning the electrical, as well as the co-propagating non-electrical, i.e., mechanical and thermal, manifestations of the AP suggested that this phenomenon has a (quasi) adiabatic character. This means that overall, there is no or very little transfer of heat, i.e., loss of useful energy, between the nerve/neuron and its surroundings during AP propagation. This inspired the idea that the “macroscopic” AP can be explained and modeled as an acoustic (i.e., density) energy pulse in the axonal membrane rather than an electrical energy wave along the membrane’s surface (Figure 1). For such a pulse, in line with thermodynamic theory, all the reported manifestations of its movement (e.g., electrical, mechanical, or thermal) follow from the second law of thermodynamics (using the Maxwell relations). Since the membrane interface is “decoupled” to some extent from its surroundings (see before), in its turn, propagation of such an acoustic pulse follows from the elastic properties of the membrane according to momentum conservation and not from the diffusion of ions (Figure 1). Thus, as put forward in the acoustic perspective, neuronal excitability, treated as an acoustic wave running in the axonal membrane interface, should be approached as a propagating thermodynamic state change, which can be studied by measuring macroscopic properties like pressure, temperature, volume, electric fields, pH, etc., during its movement [76]. More specifically, in the acoustic perspective, the state change of the interface induced by changes in environmental parameters, like electro-magnetic field, temperature, pressure, and/or pH value, assumably corresponds with a localized phase transition in the axonal membrane in which the lipid bilayer (and its other constituents) switches from its usual, i.e., unperturbed, fluid state into a slightly denser gel phase and back again while the pulse passes by (Figure 1). This reversible density change during acoustic pulse propagation in the axonal membrane could then, for instance, explain the experimentally measured swelling and subsequent shrinking and shortening of neurons during AP propagation, as reported by Tasaki and coworkers [75,76]. It would also account for the measured voltage pulse during AP propagation in terms of a changing membrane capacitance, and it can predict that heat is released when the membrane transitions from the fluid to a gel phase and is (at least partially) reabsorbed again when the membrane transitions back to the fluid phase, as observed experimentally [78]. As such, the scientists promoting the acoustic perspective claimed to have provided a theoretically satisfactory physical prediction of all established (both the electrical and the non-electrical) manifestations of the AP in terms of first principles, and this prediction is based on the laws of thermodynamics and the assumption that membrane lipids, acting together with other membrane constituents as a single macroscopic ensemble, play a fundamental role in the propagation of APs [74,76]. Overall, this means that, according to the acoustic perspective—which, vital to note here already (see Section 4), in its current form does not (yet) provide a comprehensive, mathematically formulated physical model—all measurable features of the AP, including the observed voltage change, represent different energetic aspects of a single macroscopic physical phenomenon that are predicted and, therefore, explained by thermodynamics. In this framework, as outlined by Schneider [76], “The only cause there is, is the initial trigger, but the couplings follow from reversibility and thermodynamics”.

As an unfortunate result of the introduction of the acoustic perspective, however, tensions arose between scientists committed to this novel perspective and those committed to the established bioelectric perspective (for instance, see Fox [18]). This is so because, upon a superficial reading, both perspectives offer explanations that should be taken as alternative answers to the same question, which appears to only concern the physical nature of the (electric) AP. Consequently, the acoustic perspective was viewed as a threat to the current bioelectric “paradigm” and treated as such [17]. However, considered more in depth, the thermodynamics-based acoustic account of the physical nature of neuronal excitability, in fact, describes how non-linear pulses, like APs, propagating in the axonal membrane resemble acoustic (i.e., sound) pulses propagating in an interface near a phase transition [11,75,76]. Besides invoking a completely different and relatively novel form of physics (i.e., (non-linear) acoustic physics) to explain and model the phenomenology of (neuro)biological excitability [11,76]—for instance, from a functional computational viewpoint—it is of interest to note that, following the acoustic perspective, compared to the digital (i.e., on–off) information encoded in the solely electric AP, the acoustic excitation wave is predicted to carry more information about the stimulus that caused it and to do so in a highly energy-efficient manner [79]. The potential impact of this notion may be realized in full by contemplating the earlier expressed conviction of Schoffeniels and Margineanu [49], who remarked that “there are at least two reasons to avoid the temptation of neglecting the energetics of the nerve impulse: (1) no explanation of a natural phenomenon can be accepted until it copes with the laws of thermodynamics and (2) heat dissipation sets practical limitations in high speed computers and knowing how the biological design deals with such aspects might prove to be of use”.

Moreover, if proven to be correct, the non-electric aspects of the AP contribute to information processing and neuronal signal transmission and should be treated as more than mere epiphenomena, as proposed in the bioelectric perspective [72]. We will return to this in the Section 4, but we already want to note that, although still largely based on predictions from physical theory, important claims made in the acoustic perspective are, in fact, supported by computational modeling and (sometimes contested) experimental evidence. Nevertheless, at this stage of its development, and in order to prevent any more unnecessary misunderstandings, it cannot be emphasized enough that considerably more theoretical and experimental work has to be done before it could be safely concluded that the acoustic perspective of the physical nature of neuronal excitability, as discussed in this paragraph, indeed provides a scientific explanation that is at least equivalent to or, overall, even more satisfactory than that offered by the prevailing bioelectric perspective. More importantly, however, to ensure progress, instead of competition and continuation of the current, largely unproductive dispute between the supporters of two fundamentally different scientific perspectives on the physical nature of the AP, characterized by misunderstandings, consequent communication failure, and lack of meaningful interaction, we propose another way forward. In this latter approach, interdisciplinary interaction between the two communities of scientists is to be stimulated, with the ultimate aim to develop a (fully) integrated, comprehensive biophysical explanation, understanding, and modeling of neuronal excitability. Preceding this, however, to prevent early failure and facilitate the success of such an ambitious and highly precarious interdisciplinary project, thus far unnoticed barriers of a more conceptual nature separating the two groups of scientists and blocking cooperation should be addressed and dealt with where possible (Figure 1).

## 4. Explaining the Physics of Neuronal Excitability: Towards an Interdisciplinary Perspective

The previous two sections of this paper served to highlight the (historic) background and discuss the scientific theories and concepts that guided the development of two highly divergent, if not incompatible [22], (bio)physical approaches to the explanation of the physical nature of neuronal excitability. From this, it is fair to conclude that, in its current form, the popular bioelectric perspective draws primarily from inductive, experimental, and data-informed logic and mechanistic standards of explanation, in which neuronal excitability is identified and studied as a dissipative, binary electrical wave phenomenon dependent on the voltage-controlled flow of ions through membrane-anchored ion channel proteins operating in a well-orchestrated, but individually identifiable, interactive manner (Figure 1). Treated as an insulator with constant capacitance, in this highly idealized framework, the (lipid) membrane interface is thought to only play a minor structurally supporting role in excitability [41]. It follows that the general underlying motivation leading the bioelectric perspective is that biological theories and models are best specified in terms of separate molecules, for which their mathematically described interactions have emergent, i.e., bottom-up, causal explanatory power typical of the mechanist commitment to scientific explanation [45,46]. In contrast, derived by logical deduction from first principles, i.e., the first (L1) and second (L2) law of thermodynamics, the poorly acknowledged acoustic perspective proposes that neuronal excitability is best understood as the physical manifestation of an adiabatic density (=sound) pulse propagating through an axonal membrane interface held near a phase transition (Figure 1). Accordingly, as the pulse propagates, the membrane changes in area, thickness, voltage, temperature, and other (macroscopic) thermodynamic state variables, as confirmed experimentally [76,78,80,81].

This macroscopic thermodynamics-based description of AP physics illustrates that, rather than emphasizing the importance of the behavior of individual molecular components and their local interactions, in general, top-down approaches applied to living systems, such as those exemplified in the acoustic account, focus on the specification of system-wide states as causal actors and the identification of optimality principles governing global system dynamics [82]. As expected, for the purpose of explanation, that is, providing an answer to the question of what a priori physical properties would enable the axonal membrane interface to function as a medium for the propagation of non-linear acoustic pulses, in this macroscopic thermodynamic perspective, molecular details are abstracted away from. Instead, system-wide antecedent or initial conditions are postulated that serve to describe facts or circumstances concerning the physical state of the system of interest, i.e., the axonal membrane, which, according to predictions from thermodynamic theory, must hold (i.e., identification of optimality principles) in order to allow for the transport of the excitation wave through the membrane interface (Figure 1). These antecedent conditions, for instance, include the proposition that the axonal membrane constitutes a separate thermodynamic ensemble with its own thermodynamic states (e.g., fluid or gel-like) and properties (e.g., compressibility and density) dependent on and influenced by the interaction between thermodynamic variables (e.g., pressure and temperature) (C1). Another antecedent condition in the acoustic perspective is that an initial excitation (e.g., depolarization or other perturbation) of the axonal membrane is needed to realize the liquid-to-solid phase transition, i.e., to provide the energy required to push the membrane through the phase transition and, thereby, initiate the acoustic pulse (C2). Operating collectively, these antecedent conditions, according to thermodynamic theory, constitute the essential physical membrane interface features and circumstances that permit the propagation of an acoustic pulse accompanied by reversible changes in area, thickness, voltage, and temperature (and other thermodynamic state variables), such as the phenomenon manifested in the electric and non-electric signs of the AP [76,83,84,85]. This line of reasoning—in which the phenomenon of interest, i.e., a propagating acoustic pulse in the axonal membrane, is predicted (i.e., logically derived) from the combination of proposed antecedent conditions of the axonal membrane (C) and the thermodynamic laws (L) acting on it—concurs with the requirements of the so-called covering law or deductive nomological account of explanation, originally introduced by Hempel and Oppenheim [86] and thereafter amended by other philosophers of science [87]. Commitment to this type of explanation in the acoustic perspective is, for instance, reflected in the words of Schneider [76], who argues that “For sound all the reported couplings (mechanical, electrical, chemical, optical and thermal) arise naturally as a requirement of the second law of thermodynamics (Maxwell relations, L2). Furthermore, it needs some convincing justification why perturbations (C2) of any sort within the membrane should not lead to the propagation of pulses. Since the membrane is, at least partially decoupled from the bulk (C1) … propagation follows from momentum conservation (L1) and its absence therefore violates a fundamental physical principle, which should not be taken lightly at all”. Prioritizing general physical theory and logical deduction over experimentation and inductive reasoning, following the covering-law approach, experimental verification of predictions serves primarily to sustain the validity of the claims and provide empirical content for the explanation. For this purpose, for instance, in recent years, experimental evidence has been gathered showing an intriguing resemblance between APs and acoustic pulses propagating in an artificial lipid membrane held near phase transition, including a bi-phasic pulse shape, all-or-none behavior and—addressing criticism from adherents of the bioelectric perspective on earlier claims (see further)—annihilation upon collision (for an overview of these results, see [11,23]). Moreover, preliminary data have been reported from excitable plant cells and cultured neurons demonstrating that excitation waves can be elicited by diverse physical stimuli and that their movement along the cellular membrane is accompanied first by condensation (freezing) of the membrane during the depolarization phase followed by melting (relaxation) as the membrane polarizes again [88,89].

The main idea behind interdisciplinarity in science or other academic activities has been described as an attempt to analyze, synthesize, and harmonize links between disciplines into a coordinated and coherent whole [90,91]. Thus, accepting that there is sufficient reason to support the development of an interdisciplinary, fully integrated and comprehensive account of the physical foundations of neural excitability incorporating (relevant aspects of) both the bioelectric and acoustic perspective, it is highly relevant to start with identifying obstacles that have, thus far, blocked meaningful progress in this project. In doing so, the discussion in the previous paragraphs and sections illustrates that this task, requiring interaction and cooperation between adherents to either of the two perspectives, is complicated not only by different views between the groups of scientists involved about (interpretation of theoretical and/or experimental) data and evidence or (longer term) scientific aims. In fact, it appears as if the main impediment to communication and efforts aimed at interdisciplinary integration between the two communities of scientists may be of a more fundamental, i.e., philosophical, nature. In our view, this is so because both groups are clearly committed to conflicting views of explanation that closely correspond to philosophical accounts of mechanistic and covering-law explanations (Figure 1). It is these contrasting explanatory standards rather than “mere” differences of opinion about the interpretation of data or scientific aims that block attempts at productive communication and integration between the bioelectric and acoustic perspectives on neuronal excitability. Thus, experimentalists and supporters of the bioelectric perspective in general favor a form of explanation that is based on accurate and detailed molecular-level descriptions. In contrast, the more theoretically inclined supporters of the acoustic perspective assign explanatory power primarily to generality and abstract formalizations. Consequently, each perspective construes explanatory power in a way that excludes the other [92]. Paraphrasing Green et al. [24], dealing with a comparable case in stem cell research, from the theoreticians’ (i.e., acoustic) perspective, insights based exclusively on molecular mechanistic investigations lack explanatory power because they are built on context-dependent (i.e., experimental) details and include many irrelevant details. For instance, “theoreticians” have criticized the bioelectric HH framework and model because it excludes a role for changes in membrane capacitance in determining total membrane current that is dependent on the use of the voltage-clamp technique in experiments but includes 3–4 equations that have no physical basis and rely on 4–6 fit parameters per equation [22]. On the other hand, for “experimentalists” (i.e., representing the current bioelectric perspective), abstract descriptions of dynamic principles lack explanatory power because they do not realistically investigate and describe causal, i.e., molecular, interactions [24]. For this reason, the acoustic perspective is probably negated by “experimentalists” because it does not assign any particular role to ion channel proteins (or other specific molecular entities) and is even, incorrectly, taken to deny the existence of ion channels as such (see discussion in Drukarch et al. [11]). Moreover, its extensive use of non-biological experimental models, especially artificial lipid mono- and bilayers, to experimentally substantiate its theory-based claims is not appreciated by representatives of the bioelectric perspective, who are accustomed to investigating excitability in living cells and tissues and/or materials derived therefrom [22]. Overall, similarly to the stem cell case described by Green et al. [24], this has resulted in a situation in which “From each perspective, explanations from the other do not seem merely incomplete or flawed-they seem not to be explanations at all”. To bear fruit along the road to interdisciplinary integration, therefore, a synthesis between both perspectives, in the words of Green et al. [24], “must involve a conception of explanation that allows for both general principles and specific mechanistic descriptions to bear explanatory weight”. A key step in building such an integrated conception will be for the two communities of scientists involved to recognize the legitimacy of other types of scientific explanations than the sort they aim to construct [23]. In other words, integration will only be feasible if the parties involved not only recognize but also respect the explanatory standards of both approaches. This is different from the current situation, in which each community treats its own explanatory standards as universal and mostly ignores or even mischaracterizes the other. As noted by Fagan [23], it is especially this latter approach that blocks productive interdisciplinary engagement, which requires that the groups of scientists involved develop some tolerance for explanatory norms other than those of their own research community. One way to achieve this is to look for common ground in the two varieties of explanation, allowing for complementary aims to be established (Figure 1). In this latter context, it is important to note that concerning the physical nature of neuronal excitability, although usually overlooked when discussed, the scientific goals pursued in the two perspectives are not the same. Rather, in the bioelectric perspective, a mechanist type of explanation is proposed to account specifically and, at least thus far, exclusively for the electrical properties of APs, whereas in the acoustic perspective, a covering-law type of explanation is sought to cover all the observed physical manifestations of the nerve signal in a single unified framework. Thus, both communities of scientists are using valid explanatory strategies to pursue their specific, but different, aims regarding their notion of neuronal excitability.

At the same time, however, following Fagan [23], because the ultimate goal(s), structure, and norms of the two kinds of explanation contrast sharply and, to some extent, are incompatible (e.g., inclusion vs. abstraction of specific molecular details)—albeit granting that each kind of explanation is legitimate and thereby allowing for genuine integration in the first place—to link the two communities, one of the two views of explanation will have to be given priority whilst the other will have to be accommodated it. Representing the minority view and, therefore, having the most to gain from integration, in the case of the physics of the AP, we propose that the emphasis in this effort should lie on, at least to some extent, aligning the acoustic perspective with mechanistic explanations, as used in the mainstream bioelectric perspective. From a practical point of view, one way to do so may be to first start with addressing scientific concerns voiced by “the other side”. Indeed, this was already done by the group of Matthias Schneider, who demonstrated, both theoretically and experimentally, annihilation upon collision of two non-linear acoustic pulses generated in artificial lipid membranes, using a further developed version of the original, strictly adiabatic, soliton pressure pulse framework introduced by Heimburg and Jackson [93,94]. Another way to pursue integrative progress is to explore how lacunes in the bioelectric perspective may be filled in by essential constituents of the acoustic perspective. As an example of such a complementary approach aimed at integration, it might be investigated, for instance, whether the acoustic perspective offers a valuable approach to the investigation and explanation of AP timing, a notoriously difficult-to-explain characteristic of neuronal signaling vital to our understanding not only of neuronal physiology but also of computation and cognitive processing in the brain [5,95]. In fact, at high firing rates, some cortical neurons appear to violate the HH theory in that ion channels, operating as a group in unison, open faster than predicted, allowing for a higher speed of information transfer [96]. One possible explanation of this phenomenon is that these ion channels respond together to a sudden structural change in the axonal membrane interface [97]: for instance, the arrival of a mechanical wave. The formation of such activity-dependent clusters of ion channels (and other signaling molecules) into distinct membrane microdomains known as lipid rafts has been proposed to involve a localized phase transition of membrane lipids from a (primarily) liquid to a solid state [98], pointing to a possible role for soliton-like electromechanical waves as proposed in the acoustic perspective. Interesting to note in this context is that the lowering of membrane cholesterol levels, a highly enriched component of lipid rafts essential for their formation, is accompanied by a significant reduction in AP propagation speed [99]. Further theoretical and experimental investigation of these multi-physical, particularly electromechanical, notions concerning the control of neuronal excitability could also be used to unravel the functional contribution of the lipid membrane bilayer in the increasingly recognized mechanosensitivity of different types of voltage-gated ion channel proteins involved in neuronal signaling [100]. For instance, selective ionic permeability in artificial lipid membrane layers lacking any proteins has been shown to increase considerably in the lipid phase transition regime [101], resulting in the formation of so-called lipid ion channels [102]. Reportedly (Zecchi and Heimburg, [103]), such channels share a large number of electrophysiological characteristics with regular types of mechanosensitive ion channel proteins, which, if present, may confer additional structural and/or functional properties to the axonal membrane necessary for optimal control of membrane excitability and, more particularly, AP timing and/or propagation. Thus, the development of a joint account for the initiation and propagation of neuronal signals fully integrating elements of the bioelectrical and acoustic perspectives offers a (neuro)biologically relevant topic for an interdisciplinary research program aimed at accommodating the alternative acoustic perspective with the mainstream bioelectric perspective (Figure 1). Succeeding in doing so is likely to significantly enhance scientific insight into the physical underpinnings of neuronal excitability and its role in neuronal physiology and function.

## 5. Debating the Physical Nature of Neuronal Excitability: Some Concluding Remarks

Taking in the arguments put forward in the previous sections, one may be left with the question whether sufficient evidence has been produced to propose that the (neuro)biological and/or computational biosciences are in need of a new or, at least, improved framework to investigate, explain, understand, and model the physical nature of neuronal excitability? Considering the phenomenon of neuronal excitability as the nexus between the modern scientific understanding of neuronal activity and nervous system function, this is not a trivial question to consider. Taking the primary task of science to be the building of testable explanations based (as much as possible under the prevailing circumstances) on a complete and accurate description of the phenomenon of interest, it seems safe to conclude that the available data do pose a serious challenge to the exclusive validity of the electric circuit framework of membrane excitability developed over the last 70 years or so from the ionic hypothesis of HH [10,11,12]. However, the lukewarm if not outright hostile reception by supporters of the electric framework representing the mainstream, i.e., majority, view of alternatives proposed thus far may, at least in part, have been caused by the failure of the contenders (including those suggesting a coupling of the electrical framework to non-electrical manifestations into an “extended” bioelectric perspective; see [10] to clearly outline the potential advantages of their proposals over the reigning view [18]). In fact, by highlighting and emphasizing the importance of practical instead of fundamental scientific considerations to answer the above question, critics tend to rephrase the original question and make their judgment appear to be dependent on the presentation of (at least circumstantial) experimental evidence that problems that cannot be (sufficiently) answered using the current electric framework can be addressed better and, preferentially, even be solved by consideration of the proposed alternatives [18]. However, if taken up by representatives of the acoustic perspective, these physical scientists, in contrast, have tended to emphasize the ability of their largely theory-informed approach to provide a proper explanation for the experimentally observed reversible heat production during AP propagation, which, as noted by Alan Hodgkin himself [48], is not properly accounted for in his and Huxley’s electric theory and model and, therefore, if unequivocally proven would seriously undercut the validity of their explanation [78]. Also, other shortcomings of the prevailing electric framework have been highlighted by this latter group of scientists without, however, addressing the major concerns and requirements of their opponents [74,75,76,81,84]. Not surprisingly, therefore, interested primarily in answers to their own specific research questions, these arguments from thermodynamics have failed to impress the primarily mechanism-oriented experimentalists representing the large majority of scientists operating within the mainstream bioelectric perspective on neuronal excitability. Unfortunately, as a result of this misunderstanding and misalignment of the scientific priorities and expectations of the two communities of scientists, communication failure ensued, and the original question has been left unanswered (Figure 1 [104]). Considering its importance and the potential impact of the answer, this has left the bioscientific community in an undesirable impasse. Suggestions to overcome this unwelcome situation by promoting a truly interdisciplinary research program involving scientists from both perspectives jointly addressing outstanding scientific questions of mutual interest form the essence of the solution put forward by us. Examples of possible topics to be considered as part of such a program are outlined.

However, in order to succeed with such an ambitious project, besides identification of complementary scientific aims, we have also argued that the often-implicit philosophical commitments, i.e., prioritizing mechanist- or covering-law-type explanations, of the two communities of scientists involved have to be recognized first and discussed between the participants (Figure 1). In fact, at least in our opinion, failure to address and deal with this philosophical dimension of the conflicting views on the physical nature of neuronal excitability is not only the main cause of the communication failure characterizing the controversy in the recent past but also poses the most serious threat to the much desired future development of an integrated, interdisciplinary perspective. We hope that the approach outlined by us will assist those scientists motivated to overcome the current standstill. We trust that the rewards of doing so will prove to be worth the effort.

## Data Availability

No new data were created or analyzed in this study. Data sharing is not applicable to this article.
